# Criteria for early discharge of drowning patients from the emergency department

**DOI:** 10.1111/1742-6723.70012

**Published:** 2025-02-14

**Authors:** Ogilvie Thom, Kym Roberts, Susan Devine, Richard C Franklin

**Affiliations:** ^1^ College of Public Health, Medical and Veterinary Sciences, James Cook University Townsville Queensland Australia; ^2^ Surf Life Saving Queensland South Brisbane Queensland Australia; ^3^ Sunshine Coast Hospital and Health Service Birtinya Queensland Australia; ^4^ Royal Life Saving Society— Australia Sydney New South Wales Australia

**Keywords:** drowning, emergency department, rapid discharge criteria

## Abstract

**Objective:**

Clinical factors previously shown to independently predict safe discharge were applied at ED presentation to determine whether we could identify a group of drowning patients who do not require treatment and are thus safe for rapid discharge.

**Method:**

We conducted a retrospective study of drowning presentations to EDs of the Sunshine Coast Hospital and Health Service in Queensland, Australia between 1 January 2015 and 31 December 2022. Rapid discharge criteria (RDC) were defined as a normal spontaneous respiratory rate (adjusted for age), a normal peripheral blood saturation (≥94%) on room air, an Alert measurement on the Alert, Voice, Pain, Unresponsive scale, clear chest auscultation and the absence of any requirement for oxygen or other ventilatory assistance from Emergency Medical Services. Primary outcome was the requirement for treatment (oxygen, ventilation or airway intervention).

**Results:**

Five hundred and seventy‐seven drowning presentations were included. Two hundred and fifty‐five (44.2%) patients met RDC at ED presentation. Patients meeting RDC were more likely to be younger (median 9 years, IQR 3–21 *vs* 20 years, IQR 4–44, *P* < 0.016) than those with not meeting RDC. Eight patients meeting RDC had received bystander CPR. There were no deaths in the RDC group (0/255 (0%) *vs* 17/322 (5.3%), *P* < 0.016). No patient meeting RDC required treatment (0/255 (0%) *vs* 145/322 (45.0%), *P* < 0.016).

**Conclusions:**

Drowning patients who meet rapid discharge criteria at ED presentation will not require treatment for their drowning and may be considered for discharge from the ED without further investigation or mandatory period of observation.

## Graphical abstract

Adult and paediatric drowning patients presenting to the emergency department with a normal respiratory examination do not require treatment. These patients can be safely discharged without further investigation or observation.

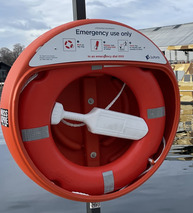

**Key findings**

- Drowning patients who meet rapid discharge criteria will not require treatment.
- This applies to adult and paediatric patients.
- Early discharge should be considered in drowning patients without other cause for admission, such as drowning associated cardiac arrest or associated trauma.

## Introduction

Drowning results in approximately 300 000 deaths globally^1^ and 500 in‐patient admissions^2^ in Australia every year. The annual number of ED attendances for drowning is likely to be more than five times the number of in‐patient admissions.^3^ Concerns regarding secondary drowning (noting that this term is no longer used) were raised at least as far back as the 19th century^4^ and have persisted well into the 21st century.^5^, ^6^, ^7^ The clinical guidelines for drowning at Children's Health Queensland,^8^ the Royal Children's Hospital in Melbourne,^9^ the New South Wales Emergency Care Institute^10^ and Perth Children's Hospital^11^ all recommend between 4 and 8 h of observation of asymptomatic drowning patients prior to discharge. The recommended timing of discharge for asymptomatic drowning patients may reflect continuing concerns regarding complications following drowning or the fact that previous studies on discharge criteria for drowning patients have been conducted at between 6^12^, ^13^ and 8^14^ h post drowning event or ED presentation. However, recent reviews have recommended discharge without further observation or investigation for asymptomatic patients.^15^ The study cited in support of this showed no mortality in 1085 drowning patients with clear chest auscultation, but 115 (11%) of these patients were also noted to have confusion.^16^

A single study that examined predictors of safe discharge at ED presentation for paediatric drowning patients found abnormal pulse oximetry readings (SpO_2_) on arrival were associated with subsequent deterioration.^17^ Normal SpO_2_ after 6–8 h in the ED has been shown to independently (OR 6.80) predict successful discharge.^18^ Other independent predictors for successful discharge include an inspired oxygen fraction of 0.21 (OR 7.05), a lack of field intervention (OR 5.10),^18^ normal ED mentation (OR 5.04) and respiratory rate (OR 2.41), as well as the absence of dyspnoea (OR 4.37).^14^ Conversely, factors independently predicting admission include the presence of respiratory distress in the ED (OR 32.7) and the auscultation of lung rales (OR 6.8–9.2).^13^

All of the published evidence on criteria for successful ED discharge in drowning has been conducted in relatively small paediatric populations,^12^, ^13^, ^14^, ^17^, ^18^ and only one has included salt water drownings.^13^ We aimed to establish whether drowning patients at ED presentation meeting rapid discharge criteria (RDC), as defined by factors previously reported to be independently associated with safe discharge, would identify a group of drowning patients who do not require treatment, in both adults and children.

## Methods

This is a secondary analysis of data from a drowning registry and the data collection tool has been published previously.^19^ All drowning patients presenting to the EDs of the Sunshine Coast Hospital and Health Service (SCHHS) between the 1 January 2015 and the 31 December 2022 were included. Drowning was defined as the presence of respiratory impairment after submersion in a fluid.^20^ There were no exclusion criteria. In 2022, the two EDs had over 140 000 presentations. Senior and junior medical staff routinely work at both sites and there is identical inpatient support (such as ICU) available. The Sunshine Coast has a population of 336 522^21^ and there are many popular surf beaches along the coast as well as public and private swimming pools, swimming holes, rivers and creeks. The Sunshine Coast is a popular destination for visitors with over eight and a half million visitor overnight stays and four and a half million visitor day visits during the 2019/2020 financial year.^22^ The present study received ethical approval and an exemption from obtaining patient consent from the Metro North Human Research and Ethics Committee (project number: 49754) and James Cook University Human Research Ethics Committee (H8104). The present study is published following Strengthening the Reporting of Observational Studies in Epidemiology (STROBE) guidelines.^23^

Patient data were collected from Surf Life Saving Queensland, Queensland Ambulance Service and SCHHS patient records and an Utstein Style for Drowning^24^ type database was used for data entry. Utilising previously reported discharge criteria, we defined RDC as the following: a normal spontaneous respiratory rate (adjusted for age),^25^ a normal SpO_2_ (>93%) on room air, a normal Alert, Voice, Pain, Unresponsive (AVPU) measurement (Alert), the absence of added chest sounds on respiratory auscultation and the absence of any requirement for oxygen or other ventilatory assistance from Emergency Medical Services (EMS). We utilised the first recorded data after ED presentation for respiratory rate, SpO_2_, AVPU and chest auscultation. Children were defined as under 16 years of age as per Children's Health Queensland guidelines.^25^ EMS treatment was obtained from EMS records. EMS included acute care and critical care paramedics from the Queensland Ambulance Service as well as aeromedical retrieval teams from LifeFlight. Patients were classified as not meeting the criteria if the data were missing.

The primary outcome, treatment, was defined as the delivery of any oxygen or other ventilatory assistance. This included simple oxygen delivery, hi‐flow nasal prongs, non‐invasive ventilation, manual ventilation and mechanical ventilation. Ancillary treatments for drowning, such as antibiotics, were excluded from this definition as was the treatment of traumatic injuries associated with the drowning event or medical conditions that precipitated the drowning event. This includes conditions such as cervical spine injuries, dislocated shoulders, intracerebral haemorrhages and intoxication. Causes of patient representation were classified as recorded in the medical notes.

All drowning occurring in tidal bodies of water (i.e. river estuaries, canals, ocean) were classified as salt water and drowning occurring in swimming pools were classified as fresh water, along with rivers (non‐tidal), creeks, swimming holes and dams. Outcomes of non‐fatal drowning were classified using the categorisation of non‐fatal drowning published by the World Health Organisation.^26^ Queensland Health statewide data were examined for any ED or hospital representation within 7 days.

Data were analysed using IBM SPPS Version 29. Continuous variables are expressed as mean and standard deviation or median and interquartile ranges depending on their distribution. Proportions are presented with 95% confidence intervals. A post hoc power analysis was performed using the overall incidence of the primary outcome (25%) and the desired incidence (0%) in the RDC group, with an alpha of 0.05. Our sample size of 255 resulted in a power of 1 to detect this difference. Relationships between categorical groups were analysed using chi‐squared analysis or Fisher's exact test when expected frequencies were below 5. A Bonferroni correction was added for multiple calculations. Means were compared using the Mann–Whitney *U* test for non‐parametric data and Student's *T* test for parametric data.

## Results

There were 577 drowning patients included in the study. Two hundred and fifty‐five (44.2%) patients met RDC. Patients meeting RDC were more likely to be younger and were less likely to be transported to the ED by EMS than patients who did not meet RDC. The majority of drownings in both groups occurred in salt water, but there was a higher proportion of fresh‐water drownings in the RDC group. Results are detailed in Table 1.

**TABLE 1:** Patient characteristics

	RDC present (*N* = 255)	RDC absent (*N* = 322)	*p* value
Age (years), median (IQR)	9 (3–21)	20 (4–44)	0.001
16 years and older	86 (33.7 ± 4.2%)	194 (60.2 ± 6.6%)	
Under 16 years	169 (66.3 ± 8.2%)	128 (39.8 ± 4.4%)	
Sex			0.042
Female (*n* = 231)	114 (44.7 ± 5.5%)	117 (36.3 ± 4.0%)	
Male (*n* = 346)	141 (55.3 ± 6.8%)	205 (63.7 ± 7.0%)	
Submersion duration (min)			
Median (IQR)	1 (1–1)	1 (1–2)	
Bystander CPR	8 (10.5%)		<0.001
Yes	8 (3.1 ± 0.4%)	68 (21.1 ± 2.3%)	
No	247 (96.9 ± 11.9%)	254 (78.9 ± 8.6%)	
Body of water			0.010
Fresh	117 (45.9 ± 5.7%)	108 (33.5 ± 3.7%)	
Salt	137 (53.7 ± 6.6%)	213 (66.1 ± 7.3%)	
Witnessed			0.001
Bystander	194 (76.1 ± 9.4%)	196 (60.9 ± 6.7%)	
Lifeguard	22 (58.6 ± 1.1%)	40 (12.4 ± 1.4%)	
Unwitnessed	33 (12.9 ± 1.6%)	76 (23.6 ± 2.6%)	
Transport to ED			<0.001
EMS	158 (62.0 ± 7.6%)	273 (84.8 ± 9.3%)	
Other	97 (38.0 ± 4.7%)	49 (15.2 ± 1.7%)	
ED ventilatory support			<0.001
Nil (*n* = 434)	255 (100 ± 12.3%)	179 (55.6 ± 6.1%)	
Nasal prongs	0 (0%)	37 (11.5 ± 1.3%)	
Hudson mask	0 (0%)	13 (4.0 ± 0.4%)	
Non‐rebreather mask	0 (0%)	14 (4.3 ± 0.48%)	
Hi‐flow nasal prongs	0 (0%)	24 (7.5 ± 0.8%)	
CPAP/BiPAP	0 (0%)	11 (3.4 ± 0.4%)	
Bag‐valve‐mask	0 (0%)	6 (1.9 ± 0.2%)	
Mechanical ventilation	0 (0%)	38 (11.8 ± 1.3%)	
Chest radiology	(*n* = 71)	(*n* = 252)	<0.001
Clear	66 (92.9 ± 11.5%)	119 (47.2 ± 6.1%)	
Unilateral oedema (*n* = 32)	2 (2.8 ± 0.4%)	30 (11.9 ± 1.3%)	
Bilateral oedema (*n* = 104)	3 (4.2 ± 0.1%)	101 (40.1 ± 4.4%)	
Consolidation/atelectasis	0 (0%)	2 (0.8 ± 0.06%)	
ED disposition			<0.001
Home	155 (60.8 ± 7.5%)	96 (29.8 ± 3.3%)	
ED SSU	89 (34.9 ± 4.3%)	83 (25.8 ± 2.8%)	
Coronary care unit	0 (0%)	2 (0.6 ± 0.1%)	
Intensive care unit	1 (0.4 ± 0.12%)	49 (15.2 ± 1.7%)	
Other inpatient unit	10 (3.9 ± 0.48%)	73 (22.7 ± 2.5%)	
Inter‐hospital transfer	0 (0%)	11 (3.4 ± 0.4%)	
Died in ED	0 (0%)	8 (2.5 ± 0.3%)	
Status at hospital discharge			<0.001
Died	0 (0%)	17 (5.3 ± 0.6%)	
Severe disability	0 (0%)	2 (0.6 ± 0.1%)	
Moderate disability	1 (0.4 ± 0.1%)	7 (2.2 ± 0.2%)	
No morbidity (*n* = 550)	254 (99.6 ± 12.3%)	296 (91.9 ± 10.1%)	
7 day represent			0.299
Yes	5 (2.0 ± 0.2%)	12 (3.7 ± 0.4%)	
No	250 (98.0 ± 12.1%)	310 (96.3 ± 10.6%)	

BiPAP, bi‐level positive airway pressure; CPAP, continuous positive airway pressure; CPR, cardiopulmonary resuscitation; D/C, hospital discharge; ED SSU, emergency department short stay unit; ED, emergency department; EMS, emergency medical services; IPU, inpatient unit; RDC, rapid discharge criteria.

No patient with a RDC required treatment while in the ED, nor after admission to either the ED SSU or an inpatient unit. The majority of patients who did not meet RDC also (179/322, 55.6%) did not require treatment in the ED or after admission (140/226, 61.9%).

There were 17 deaths, all in patients who did not meet RDC. Ten patients were discharged with a documented morbidity, nine where RDC were absent and one where the RDC was present. The single patient in the RDC group discharged with morbidity had morbidity secondary to an intracerebral haemorrhage, the likely precipitant of the drowning incident.

There were eight patients meeting RDC group who had received bystander CPR. There were two males and six females, three patients were adults and five were children (range 3–48 years). The majority of these patients (*n* = 6) drowned in fresh water and the duration of drowning was ranged between 1 and 5 min (*n* = 3). The duration of the bystander CPR was only documented twice (1–3 min); however, all responded prior to arrival of EMS. Four of these patients were discharged directly home, two were admitted to the ED SSU, one to an inpatient unit and one to the paediatric ICU. All four admitted patients were successfully discharged after a period of observation without need for treatment. None of these eight patients represented in the following 7 days. Radiological imaging of the chest was obtained in 323 (56.0%) patients. The proportion with normal findings was twice as high in the RDC group (93.0%) compared to those that do not meet RDC (47.2%). Patients with bilateral or unilateral oedema on their imaging were more likely to require treatment (*X* ^2^ = 269.52, df 10, *P* = 0.007) than those with normal findings; however, 33/136 (24.3 ± 12.7%) patients with radiological pulmonary oedema did not require treatment. Conversely 23/185 (18.4 ± 10.8%) of the patients with normal radiological findings required treatment. Six patients who required treatment did not undergo radiological imaging.

Table 2 presents the performance of the individual criteria in detecting a population of drowning patients that does not require treatment. The Rapid Discharge Criteria had a specificity of 100% in detecting patients that did not require treatment. The likelihood ratio (LR) for not requiring treatment in the ED was highest for patients with a normal SpO2 and those that had not received treatment from EMS. All individual components of the RDC had confidence intervals for the LR above 1. A LR for the RDC could not be calculated because of the 100% specificity.

**TABLE 2:** Performance of RDC criteria for patients not requiring ED treatment

	Nil ED Rx	Received Rx	Sensitivity (95% CI)	Specificity (95% CI)	Likelihood ratio (95% CI)
Meets RDC	255	0	59.0 (54.2–63.7%)	100 (97.5–100%)	—
Does not meet RDC	177	145			
Normal RR	356	42	82.4 (78.5–85.9%)	71.0 (62.9–78.3%)	2.85 (2.20–3.68)
Abn RR	76	103			
Normal SpO_2_	409	12	94.7 (92.2–96.6%)	91.7 (86.0–95.7%)	11.4 (6.7–19.7)
Abn SpO_2_	23	133			
Normal chest exam	345	38	79.9 (75.8–83.6%)	73.8 (65.9–80.7%)	3.05 (2.31–4.02)
Abn chest exam	87	107			
Alert	430	98	99.6 (98.3–99.9%)	32.4 (24.9–40.7%)	1.47 (1.32–1.65)
Not Alert	2	47			
Nil EMS Rx	364	14	84.3 (80.5–87.6%)	90.3 (84.3–94.6%)	8.73 (5.30–14.38)
EMS Rx	68	131			

Abn, abnormal; ED, emergency department; EMS, emergency medical services; RDC, rapid discharge criteria; RR, respiratory rate; Rx, treatment; SpO_2_, peripheral oxygen saturation.

There were 18 patient representations to the hospital within 7 days of discharge. Five patients meeting RDC represented, all of which were unrelated to the drowning injury. Thirteen patients where RDC was absent represented. Three were for a planned review, three patients because of ongoing parental concerns and seven patients with issues unrelated to the drowning. These were often related to precipitating illness' for the drowning event or associated traumatic injuries. No representation resulted in any patient receiving treatment for drowning or requiring same day in‐patient admission.

## Discussion

The requirement for mandatory periods of observation prior to the ED discharge of drowning patients meeting RDC at presentation is not supported by our results. No patient meeting RDC required treatment in the ED, in SSU or after admission to an in‐patient unit. This includes the five RDC patients with radiographic evidence of pulmonary oedema and eight patients who had received bystander CPR. Our definition of a RDC appears to be conservative indicator of the need for treatment, as over 50% (177/322) of patients who did not meet RDC also did not require treatment in the ED or in‐patient unit either. Drowning patients without other cause for ED assessment who meet RDC do not require treatment, and as such they do not require admission for further observation or investigation. Early discharge from the ED may help decrease ED bed pressures, allowing SSU and in‐patient beds to be better utilised for patients more likely to require treatment.

Patients meeting RDC were more likely to be younger when compared with those not meeting RDC. The younger median age in the RDC group may possibly reflect ED presentations driven by parental concern with younger children.

All but one of the patients meeting RDC were discharged from the hospital without morbidity. This highlights the value of the RDC as a means of measuring the severity of the drowning injury. The fact that morbidity in the patient with a RDC was secondary to the intracerebral haemorrhage that likely precipitated the drowning event again reinforces the conservative nature of the RDC assessment in relation to severity of drowning. The criteria used for the RDC assessment were evidence based, utilising clinical parameters that had been shown to be independently associated with ED discharge of drowning patients.^13^, ^14^, ^17^, ^18^

Despite the conservative nature of the RDC assessment, eight patients with a RDC had undergone bystander resuscitation for drowning associated cardio‐respiratory arrest (DACA). All eight had established Return of Spontaneous Circulation (ROSC) prior to the arrival of EMS. Four were well enough to be discharged home directly from the ED. Rapid and complete recovery from DACA has been documented elsewhere, with 11/33 (33%) paediatric drowning patients discharged home directly from the ED having received bystander CPR.^27^ We would strongly recommend against the early discharge of patients who have received bystander CPR, whether or not they meet RDC. Pearn describes the deterioration of five seemingly normal children after arrival at hospital.^7^ The deterioration took place up to 48 h after admission.^7^ Four of these children had received bystander CPR, three for an extended period (15–60 min). Although the present study predates the use of pulse oximetry, the cautionary tale in patients who have received bystander CPR for DACA should continue to be respected.

There was no difference between those meeting and not meeting RDC in the number of patients who represented to the ED in the 7 days following discharge. No patient who represented from either group required oxygen, or same day in‐patient admission. We were unable to access general practice (GP) records for representation so our results may underestimate the true figure. Patients requiring urgent assessment and treatment are typically transferred to the hospital from the GP *via* the ED and would subsequently have been found using our methodology. Thus, we feel our conclusion that no patient represented within 7 days requiring treatment for drowning is valid.

Despite scant evidence supporting its existence, the term secondary drowning persists both in the medical literature^6^ as well as public perception and social media. There is no doubt drowning patients can deteriorate after their injury, as with any other form of trauma. However, deterioration after presenting to the ED with a normal examination is rarely documented. Brennan reported respiratory complications developing in two drowning patients who had normal vital signs at ED presentation.^17^ One of these patients had an abnormal cardiopulmonary exam and would not have met RDC in our study. The other patient with normal vital signs and a normal cardiopulmonary examination, developed intermittent grunting within an hour of presentation, but required no treatment.^17^ Other reports document deterioration in drowning patients who had abnormal vital signs or concurrent illnesses.^5^, ^6^, ^28^

Our study has the advantages of being the first study specifically investigating ED discharge criteria to include both adult and paediatric patients, and only the second to include patients who drowned in salt water. It also has the advantage of a larger sample size. Ultimately, the purpose of monitoring patients in the ED, SSU or in‐patient unit is to detect those that will require treatment. Our findings will allow improved selection of drowning patients at increased risk of needing treatment, in both adults and children.

## Limitations

This is a single centre retrospective study. Our search strategy to identify all drowning patients presenting to the SCHHS, although exhaustive,^19^ is not fail proof. As previously reported,^3^ the documentation regarding duration of submersion, the most important prognostic factor in drowning,^29^ is poor and may have impacted our results. The RDC criteria were derived from previous studies and were not independently derived. The study was conducted in a sub‐tropical climate and may not reflect drownings in cold water. Finally, radiological findings were sourced from the original reports, not from a blinded reporting.

## Conclusion

Drowning patients meeting the rapid discharge criteria of a normal age‐adjusted respiratory rate, normal SpO_2_ on room air, a clear chest examination, who are alert and have not required oxygen or ventilatory assistance from EMS do not require treatment. Further investigation or mandatory admission for observation do not appear to be required in this group.

## Data Availability

The data that support the findings of the present study are available on request from the corresponding author. The data are not publicly available because of privacy or ethical restrictions.
